# Bread Wheat (*Triticum aestivum* L.) Grain Protein Concentration Is Related to Early Post-Flowering Nitrate Uptake under Putative Control of Plant Satiety Level

**DOI:** 10.1371/journal.pone.0149668

**Published:** 2016-02-17

**Authors:** François Taulemesse, Jacques Le Gouis, David Gouache, Yves Gibon, Vincent Allard

**Affiliations:** 1 INRA, UMR 1095 Génétique, Diversité et Ecophysiologie des Céréales, 5 chemin de Beaulieu, F-63100 Clermont-Ferrand, France; 2 Université Blaise Pascal, UMR 1095 Génétique, Diversité et Ecophysiologie des Céréales, F-63178 Aubière Cedex, France; 3 Arvalis-Institut du Végétal, Station Expérimentale de Boigneville, F-91720 Boigneville, France; 4 INRA, UMR 1332 Biologie du Fruit et Pathologie, 71 avenue Edouard Bourlaux, F-33882 Villenave d’Ornon Cedex, France; University Paris South, FRANCE

## Abstract

The strong negative correlation between grain protein concentration (GPC) and grain yield (GY) in bread wheat complicates the simultaneous improvement of these traits. However, earlier studies have concluded that the deviation from this relationship (grain protein deviation or GPD) has strong genetic basis. Genotypes with positive GPD have an increased ability to uptake nitrogen (N) during the post-flowering period independently of the amount of N taken up before flowering, suggesting that genetic variability for N satiety could enable the breakage of the negative relationship. This study is based on two genotypes markedly contrasted for GPD grown under semi-hydroponic conditions differentiated for nitrate availability both before and after flowering. This allows exploration of the genetic determinants of post-flowering N uptake (PANU) by combining whole plant sampling and targeted gene expression approaches. The results highlights the correlation (r² = 0.81) with GPC of PANU occurring early during grain development (flowering–flowering + 250 degree-days) independently of GY. Early PANU was in turn correlated (r² = 0.80) to the stem-biomass increment after flowering through its effect on N sink activity. Differences in early PANU between genotypes, despite comparable N statuses at flowering, suggest that genetic differences in N satiety could be involved in the establishment of the GPC. Through its strong negative correlation with genes implied in N assimilation, root nitrate concentration appears to be a good marker for evaluating instantaneous plant N demand, and may provide valuable information on the genotypic N satiety level. This trait may help breeders to identify genotypes having high GPC independently of their GY.

## Introduction

Grain yield (GY) and grain protein concentration (GPC) are two major breeding objectives in wheat, as these traits are the dominant determinants of the economic value of the harvested product. GPC influences price, in particular because of its impact on the rheological qualities of the flour [1,2]. It is also a necessary quality criterion for wheat to be eligible for export. However, there is a strong negative relationship between GY and GPC [3–7] and this presents a major obstacle to the simultaneous improvement of these two traits in breeding programmes. In most developed nations, including in Europe, GY increased greatly during the second half of the 20^th^ century [6,8]. This gain was the combined result of improved genotypes obtained through breeding and improved management practices. Unfortunately, the global rise in GY has been associated with a concomitant decrease in GPC [9,10]. Nevertheless, as stated by Simmonds [3], breeding programs focusing on GPC would have been counter-productive because the economic cost of the associated GY penalty would have far exceeded the economic benefit of the protein gain. The classical agronomic strategy for achieving high GY coupled with a good level of GPC is to grow varieties having high GY potential and then to boost their GPC through a protocol in which the final fertiliser application is delayed to just before heading. However, as the global valorisation of fertilizer inputs is estimated between 50% and 60% in high potential conditions [11], this approach is now being questioned in the context of reduced-input agriculture, due to the large economic and ecologic cost of excessive mineral fertiliser usage [12].

A new strategy to counteract the negative relationship between GY and GPC, without compromising either of these two traits was proposed by Monaghan *et al*. [5]. This originates in the observation that some genotypes deviate from the linear regression of GPC on GY, either positively or negatively. This deviation, called the Grain Protein Deviation (GPD), has a strong genetic basis [7,10] and may thus provide an alternative selection criterion for simultaneously improving GY and GPC.

Grain N may originate from N taken up either before or after flowering. N taken up before flowering is stored in the vegetative organs but later, during their senescence, is remobilised into the grain. Genotypic differences have been detected for the fraction of grain N originating from remobilisation, both through variation in the amount of N already present in the plant at flowering [13] and through variation in the N remobilisation efficiency [14,15]. N taken up by the plant during the post-flowering period can account for between 5 and 40% of total grain N under field conditions [14,16,17]. The relative contribution of this later N source to grain N is strongly influenced by the environment–especially the availabilities of soil N and water, but also to a lesser extent by genotype [18]. Under controlled conditions where environmental constraints are minimised, wheat has the ability to take up N until near grain maturity [19–22]. However, under field conditions, pre-flowering and post-flowering N uptakes are negatively correlated [5,17,18,23]. This negative relationship may be explained by the presence in the soil of a finite amount of available N that can be absorbed either before or after flowering.

It has been shown that post-flowering N uptake (PANU) has a strong impact on GPC [5,14,24–26]. In a growth chamber, under post-flowering conditions where N is non-limiting, PANU may completely meet grain need for N [27]. Although the physiological basis of the GPD remains unclear, Bogard *et al*. [23] demonstrated that it was highly correlated with genotypic capacity to absorb N during the post-flowering period independently of the amount of N taken up before flowering. This leads to the hypothesis that genetic variability for GPD could be associated with variations in satiety for N. The positive GPD could thus be associated with genotypes having an increased ability to take up N during the post-flowering period despite a high N uptake before flowering. PANU is hypothesised to be both linked to plant N status at flowering and to the strength of the demand for N exerted by the growing grain [27]. Such internal regulations of N uptake could represent a potential node of genetic variability that might explain the increased ability of some genotypes to capture N after flowering in a way that was independent of the level of N uptake realised before flowering [23].

Under aerobic soil conditions (classically the case for wheat), N is principally taken up as nitrate (NO_3_^-^) [28]. Two main families of root transporters are involved in plants, and these are differentiated by their affinity for NO_3_^-^. The first is a high-affinity transport system (HATS), coded by NRT2 family genes, and the second is a low-affinity transport system (LATS) coded by NPF genes [29], formerly known as NRT1 family genes [30–33]. In *Arabidopsis thaliana*, it has been shown that the involvement of the two transport systems in NO_3_^-^ uptake is dependent on the NO_3_^-^ concentration in the external medium. LATS predominates when the NO_3_^-^ concentration in the medium is high (>1 mM), while HATS predominates when NO_3_^-^ concentration is low (<1 mM) [31]. Unlike the model species, information on wheat *NRT* genes is limited. Until recently, only one gene belonging to the NRT1 family had been studied [34]. This gene exhibit a high degree of homology to OsNRT1.1 from Oriza sativa, whose nitrate transport function has been confirmed [35]. A more recent study has however proposed 16 genes as putative homologs to the *A*. *thaliana* NRT1 family genes [36]. Similarly, *TaNRT2*.*1* is the only gene belonging to the NRT2 family which has so far been characterised in wheat [37]. This gene is hypothesised to play a central role in wheat post-flowering N uptake [27]. The major implication of HATS in wheat NO_3_^-^ uptake independently of NO_3_^-^ concentration in the external medium has also been suggested in recent studies [38,39], although Pang *et al*. [40] observed a significant involvement of LATS at the tillering stage.

Progress in the understanding of the mechanisms underlying N uptake and N assimilation opens the possibility of understanding the genetic variability for complex mechanisms linked to N uptake, such as GPD, at the molecular scale. To an applied perspective, such knowledge could provide the ability to select varieties with increased capacity to valorise late fertiliser inputs into grain proteins. The objective of this study was to characterise the physiological and molecular markers of GPD, using two genotypes (cvs. Récital and Renan) which contrast markedly with respect of this trait. Employing a semi-hydroponic culture method and closely controlled conditions, the experimental approach allowed fine control of NO_3_^-^ availability and testing for the effects of post-flowering NO_3_^-^ availability on plants having contrasting N statuses at flowering. The study includes physiological measurements, gene expression quantification, and NO_3_^-^ assays at four developmental stages distributed between flowering and maturity.

## Material and Methods

### Plant material

Two winter wheat (*Triticum aestivum* L.) genotypes were used in this study, the cvs. Récital and Renan. These were chosen for their strongly contrasting GPD performance found in multi-environment trials [10,23] associated with closely comparable grain yields and similar earliness. Both are semi dwarf *Rht-B1b* [15]. At comparable levels of GY, Récital has a GPC which is about 1.5% lower than Renan [10,23,41,42]. In this genotype pairing, Récital represents the GPD negative partner (GPD-) and Renan the positive one (GPD+).

Calibrated grains (55 mg ± 5 for Récital and 65 mg ± 5 for Renan, according to their respective mean thousand kernel weight values) were sown in germination trays filled with compost and placed in a heated greenhouse (20°C) for two weeks. Seedlings were subsequently vernalised for six weeks in a growth chamber (6°C, 8 h photoperiod, light intensity 350 μmol PAR m^-2^s^-1^). After vernalisation, they were transplanted to PVC tubes (7 cm diameter, 60 cm high) filled with a perlite:sand substrate (1:1, v:v) for semi-hydroponic culture, with two plants per tube. A total of 256 tubes (512 plants) was set up for each genotype. Tubes were placed vertically in eight containers (four containers per genotype, container area 0.49 m²) at a density of 64 tubes per container, each container containing eight rows of eight tubes. This configuration represents a cover density of 260 plants m^-^², which is comparable to that under field conditions under local agronomic practice. To avoid any “edge effect” on plants located on the outer edges of containers, appropriate shading nets were positioned around each container. These shading nets, positioned at the canopy height, were regularly raised during plant development.

Plants were then placed in a growth chamber under a long-day photoperiod (16 h light at 20°C, 8 h dark at 18°C, light intensity 650 μmol PAR m^-2^s^-1^). Each tube was fitted with its own automated micro-irrigation system which provided nutrient solution at a rate of 66 ml per 3 h. Nutrient solution composition was adapted from Castle and Randall [43] (S1 Table). Before flowering, the nutrient solution contained either 4 or 10 mM of NO_3_^-^ to create two pre-flowering N treatments which we will refer to as N4 and N10, respectively. During the pre-flowering period, two containers of each genotype were exposed to the N4 pretreatment, and two to N10.

From flowering and for the remainder of the post-flowering period, the nutrient solutions were reassigned among the containers so that one container from the N4 pretreatment and one from N10, were now exposed to low NO_3_^-^ availability (4 mM of NO_3_^-^). Meanwhile, the other two containers (one from the N4 pretreatment and one from N10) were now exposed to high NO_3_^-^ availability (10 mM of NO_3_^-^). This created the two post-flowering N treatments, LN and HN, respectively. The factorial design allowed independent observation of the effects of low and high NO_3_^-^ availability in both the pre- and post-flowering periods. For each genotype, it created the four treatment combinations: N4-LN, N4-HN, N10-LN and N10-HN. The homogeneity of environmental parameters within the growth chamber (temperature, humidity, light intensity) was beforehand validated, and particular care was taken concerning preparation of substrate and precision of each individual micro-irrigation system.

### Sampling protocol

Four destructive samplings were carried out during the post-flowering period. The first took place one day after flowering (GS65, [44]), and the other three at GS65+250 degree-day (DD), GS65+450 DD and maturity (GS92). Each sampling was carried out between one and two hours after lighting. At each sampling date, plants were harvested by rows from the outside to the inside of the container.

### Physiological measurements

Physiological measurements were carried out on five biological replicates at the four sampling dates, the two pooled plants of each tube being considered as a single replicate. Plants were each divided into six fractions: root, stem, green laminae, senescent laminae, grain and chaff. Only at GS65, spikes were not divided into grain and chaff. After fractioning, areas of green laminae were measured (LI-3100 area meter, LI-COR, Lincoln NE, USA) and spike numbers were counted. The different fractions were then oven-dried at 80°C for 48 h before dry weight (DW) measurements. Samples were subsequently ground using a ball mill, and total N concentrations were measured by the Dumas combustion method using a Flash EA 1112 Series CNS analyser (ThermoFisher Scientific, Waltham MA, USA). GPC was estimated as grain N x 5.7.

Nitrogen Nutrition Index (NNI) was calculated as the above-ground N concentration divided by a critical plant N concentration, defined as the minimum N concentration needed for maximum growth rate [45]. The critical plant N concentration was calculated using the equation described for winter wheat in Justes *et al*. [46]. PANU was calculated as the difference in total plant N between GS92 and GS65, and early PANU as the difference in total plant N between GS65+250 DD and GS65. Remobilisation of N was calculated as the difference between total vegetative N (total plant N, minus grain N) between GS92 and GS65.

### Preparation of root samples for NO3- concentration and gene expression analysis

Nitrate concentration and gene expression analyses were carried out for the first three sampling dates only, samples collected at maturity exhibited an advanced state of senescence. The same samples were used to assay NO_3_^-^ concentration and gene expression. Analyses were carried out on three biological replicates, the two pooled plants from each tube were considered a single replicate. Roots were separated from the aerial parts, washed free of substrate residue with water, and frozen in liquid nitrogen. Root samples were ground while still frozen using a ball mill, and stored at -80°C pending analysis.

### Metabolite measurements

Nitrate concentration measurements were carried out at the Bordeaux INRA Metabolome Platform (https://www.bordeaux.inra.fr/umr619/RMN_index.htm; Bordeaux, France) on sub-samples of root frozen powder (20 mg), using the spectrophotometric method described in Cross *et al*. [47].

### qRT-PCR analyses

Total RNA was extracted from sub-samples of frozen root powder (100 mg) using the Nucleomag 96 RNA kit (Macherey-Nagel, Düren, Germany) on the Biosprint 96 (Qiagen, Hilden, Germany). Total RNA was subsequently purified with the NucleoSpin 96 RNA kit (Macherey-Nagel). This purification step allowed a complete elimination of both protein residues and residual genomic DNA contamination, with another DNase digestion step. Reverse transcription was carried out on 1 μg of RNA with the iScript Select cDNA synthesis kit (Bio-Rad, Richmond, CA, USA). Steps of extraction, purification and reverse transcription were carried out according to the manufacturer’s instructions.

The primer pairs used to quantify expression levels of genes coding for two root nitrate transporters *TaNRT1* (GenBank AY587264) and *TaNRT2*.*1* (GenBank AF332214.1), the nitrate reductase *T*a*NR* (whose partial sequence comes from Boisson *et al*. [48]) and the glutamine synthetase 2 *TaGS2* (GenBank DQ124212.1) have been described in Taulemesse *et al*. [27]. The specificities and the efficiencies of the primer pairs had been validated previously.

Quantitative real-time experiments were carried on the Light Cycler 380 (Roche, Indianapolis, IN, USA) with the LightCycler 480 SYBR Green 1 Master Kit. Reactions were made with amounts of 12.5 ng cDNA for *TaNR* and *TaGS2*, or 31.25 ng cDNA for *TaNRT1* and *TaNRT2*.*1* because of lower expression levels observed for the two latter genes. PCR reactions were cycled for 10 min at 95°C followed by 45 cycles of 95°C for 10 s, 60°C for 15 s and 72°C for 15s. A melting curve was analysed at the end of each assay to ensure that single products were amplified. Relative expression was determined using the ∆CT method corrected for primers efficiency [49], and results were normalised to the expression of two housekeeping genes, *Ta54280* and *Ta54948*, selected from Paolacci *et al*. [50], whose expression stability had already been validated under our experimental conditions.

### Statistical analyses

Statistical analyses were carried out using R v2.15.1 [51] after conversion to a per-square-meter basis based on tube surface area. Graphics were drawn using SigmaPlot v8.0.

## Results

### Plant N status at flowering

Two contrasting N treatments were imposed on each of the two cvs. Renan and Récital before flowering (N4 and N10), to obtain plants having distinct N statuses at the flowering stage.

For both Renan and Récital, spike number per square meter, green laminae area, whole plant dry weight, whole plant N concentration, whole plant N and NNI were all strongly impacted by the pre-flowering N treatments (p<0.001) (Table 1). However, the N response of the two genotypes differed for some traits. Both Renan and Récital showed similar spike numbers under N10 condition, and again under N4 conditions. The mean decreases in spike number in response to reduced N were about 24% and 33%, for Renan and Récital respectively. Green laminae area was similar for the two genotypes across the two pre-flowering N treatments (p = 0.27), but a significant interaction term between genotype and N treatment (p = 0.02) revealed that Récital was more affected by the N4 treatment than Renan. The decrease in green laminae area between N10 and N4 was about 21% for Renan, and 57% for Récital. Whole plant dry weight responded to N deficiency in both genotypes, with an average decrease between N10 and N4 of 32% for Renan and 38% for Récital, but biomass was significantly higher in Renan than Récital across N treatments (p<0.001). The opposite result was observed for whole plant N concentration, with significantly lower levels for Renan than for Récital (p<0.001), despite similar responses to N deficiency with decreases of plant N concentration of 30% for Renan, and 29% for Récital. Total plant N showed a low significant difference between the two genotypes (p<0.05). The average decrease in total plant N in response to N deficiency was 53% for Renan, and 58% for Récital. Lastly, NNI levels were similar in the two genotypes under both N4 and N10 conditions (Table 1). Initially, the NNI index was developed to assess plant N status under field conditions [45], where a value less than ‘1’ indicates N deficiency [46]. Here, our NNI values were always higher than ‘1’, which suggests this index is not well-suited to the semi-hydroponic conditions of this study. Nevertheless, the results do confirm that the pre-flowering N treatments led to contrasting N statuses at flowering with average NNI values for the N10 and N4 treatments of 1.82 and 1.12, respectively.

**Table 1:** Spike number, green laminae area, plant dry weight, plant N concentration, total plant N and Nitrogen Nutrition Index (NNI) at flowering for the two genotypes at two contrasting pre-flowering N treatments. Values are the means of sixteen biological repetitions ± 1 standard error (SE) for Plant dry weight, plant N concentration and total plant N, and the means of ten biological repetitions ± 1 standard error (SE) for spike number, green laminae area and NNI. Statistical groups are given by post-ANOVA Tukey HSD test for α = 0.05.

Genotype	N treatment	Spike number (per m^-2^) ±SE	Green laminae area (m² m^-2^) ±SE	Plant dry weight (g m^-2^) ±SE	Plant N concentration (%DW) ±SE	Total plant N (g m^-2^) ±SE	NNI ±SE
Récital	N4	653 ±34 *c*	4.8 ±0.4 *c*	1152 ±83 *d*	1.93 ±0.11 *b*	21.1 ±0.8 *d*	1.10 ±0.04 *b*
	N10	971 ±66 *a*	11.3 ±0.6 *a*	1868 ±64 *b*	2.73 ±0.05 *a*	50.5 ±1.1 *b*	1.82 ±0.03 *a*
Renan	N4	735 ±53 *bc*	8.3 ±0.5 *b*	1555 ±67 *c*	1.77 ±0.06 *b*	27.0 ±0.6 *c*	1.14 ±0.02 *b*
	N10	970 ±94 *ab*	13.0 ±1.6 *a*	2292 ±88 *a*	2.52 ±0.09 *a*	57.6 ±2.4 *a*	1.83 ±0.12 *a*
Genotype	N treatment	Spike number (per m^-2^) ±SE	Green laminae area (m² m^-2^) ±SE	Plant dry weight (g m^-2^) ±SE	Plant N concentration (%DW) ±SE	Total plant N (g m^-2^) ±SE	NNI ±SE
Récital	N4	653 ±34 *c*	4.8 ±0.4 *c*	1152 ±83 *d*	1.93 ±0.11 *b*	21.1 ±0.8 *d*	1.10 ±0.04 *b*
	N10	971 ±66 *a*	11.3 ±0.6 *a*	1868 ±64 *b*	2.73 ±0.05 *a*	50.5 ±1.1 *b*	1.82 ±0.03 *a*
Renan	N4	735 ±53 *bc*	8.3 ±0.5 *b*	1555 ±67 *c*	1.77 ±0.06 *b*	27.0 ±0.6 *c*	1.14 ±0.02 *b*
	N10	970 ±94 *ab*	13.0 ±1.6 *a*	2292 ±88 *a*	2.52 ±0.09 *a*	57.6 ±2.4 *a*	1.83 ±0.12 *a*

### Impact of N treatments on agronomic traits at maturity

At flowering, plants from the N4 and N10 pre-flowering treatments were divided into two identical groups. One group was exposed to a low-N post-flowering treatment (LN), and the other to a high-N treatment (HN). This factorial design allowed us to record the behaviours of initially high- and low-N status plants under high- and low-N conditions during the post-flowering period. At maturity, four key agronomic traits were recorded to characterise the performances of the two genotypes. The traits were: GY, grain N concentration, grain N yield, and N harvest index (Table 2).

**Table 2:** Grain yield, grain N concentration, total grain N and N harvest index at maturity for the two genotypes studied under four N treatments. Values are the means of five biological repetitions ± 1 standard error (SE). Statistical groups are given by post-ANOVA Tukey HSD test for α = 0.05.

Genotype	N treatment	Grain yield (g m^-2^) ±SE	Grain N concentration (%DW) ±SE	Total grain N (g m^-2^) ±SE	N Harvest Index ±SE
Récital	N 4-LN	527.4 ±62.3 *d*	2.62 ±0.19 *ab*	13.47 ±1.33 *e*	0.41 ±0.02 *b*
	N4-HN	778.6 ±133.7 *cd*	2.65 ±0.11*ab*	20.06 ±2.74 *de*	0.46 ±0.04 *b*
	N10-LN	1531.6 ±116.5 *a*	2.31 ±0.06 *b*	35.14 ±1.95 *abc*	0.49 ±0.04 *b*
	N10-HN	1384.0 ±153.4 *ab*	2.33 ±0.04 *b*	32.25 ±3.72 *c*	0.43 ±0.03 *b*
Renan	N 4-LN	1281.4 ±80.9 *ab*	2.68 ±0.03 *ab*	34.29 ±2.01 *bc*	0.66 ±0.05 *a*
	N4-HN	1016.6 ±77.4 *bc*	2.64 ±0.11 *ab*	26.51 ±1.35 *cd*	0.50 ±0.05 *b*
	N10-LN	1576.3 ±72.5 *a*	2.81 ±0.08 *a*	44.28 ±2.52 *ab*	0.62 ±0.03 *a*
	N10-HN	1502.8 ±81.4 *a*	3.04 ±0.10 *a*	45.41 ±1.62 *a*	0.48 ±0.03 *b*

The semi-hydroponic growth conditions led to GY values of between 527 and 1576 g DW m^-2^ (Table 2). As expected, GY was positively affected by pre-flowering N treatments in both Récital and Renan (p = <0.001) but no significant effect of the post-flowering N treatments was detected (p = 0.42). Combining the results across all treatments, Renan exhibited a higher GY than Récital (p<0.001). A significant interaction between genotype and pre-flowering N treatment (p<0.001) showed that the GY for Récital was more affected by the low-N pre-flowering treatment (N4) than that for Renan.

Grain N concentrations varied between 2.3% and 3.0% (Table 2), these corresponding to high levels of GPC (13.2% and 17.3% respectively). Interestingly, the Anova revealed no significant effect of post-flowering N treatment on grain N concentration, as previously observed for grain yield. However, there was a strong genotype effect (p<0.001) and a significant interaction between genotype and pre-flowering N treatment (p<0.001). Hence, Renan had a higher grain-N concentration than Récital, which resulted principally from strong differences observed between the two genotypes when submitted to N10 pre-flowering treatment. An interesting observation was the opposite-going response of the two genotypes to the pre-flowering N treatments, revealed in the significant interaction term. Here, an increase in pre-flowering N availability led to a decrease in grain N concentration in Récital, but to an increase in Renan.

Total grain N was influenced mostly by GY as the latter trait varied more in response to N availability. Analysis of total grain N showed a significant genotype effect (p<0.001) and pre-flowering N treatment effect (p<0.001). The two genotypes both responded positively to high pre-flowering N availability, although when all treatments were combined, Renan had a higher grain N yield, than Récital.

The N harvest index (NHI) provides information on the distribution of N between the vegetative organs and the grain. Interestingly NHI was significantly influenced by genotype (p<0.001), by post-flowering N treatment (p<0.001) and by the interaction between genotype and post-flowering N treatment (p<0.001). Renan had a higher NHI than Récital, due to the high NHI levels recorded in the low-N post-flowering treatment (LN). The significant interaction term between genotype and post-flowering N treatment reveals that the increase in NHI observed for the post-flowering LN treatment in Renan was not apparent in Récital.

### Relations between grain yield and grain N concentration

**Fig 1:**
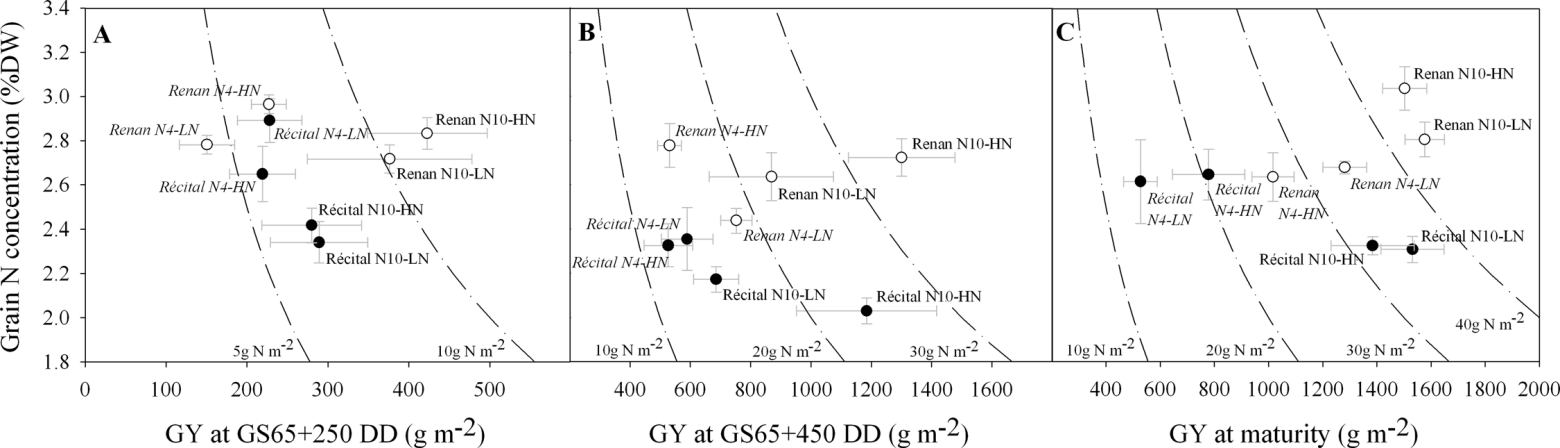
Relations between grain yield and grain N concentration at three post-flowering developmental stages. Data were collected at GS65+250 DD (A), GS65+250 DD (B) and at maturity (C) for Récital (black circles) and Renan (white circles) exposed to two contrasting pre-flowering nitrate treatments N4 (italic labels) and N10 (regular labels) combined with two contrasting post-flowering nitrate treatments LN and HN. Dotted lines are iso-grain N yield. Values are the means of five biological replicates ± 1 standard error.

The relationship between grain yield and grain N concentration is at the basis of the calculation of GPD. This study does not allow GPD to be calculated *stricto sensu* because it considers only two genotypes. Nevertheless the graphical representation of the relationship between GY and grain N concentration at maturity (Fig 1C) does offer a good visualisation of both genotypes and N treatment effects. In particular, the causes for Renan’s higher total grain N appears clearly. While this was caused by a higher GY than Récital in the case of the low-N pre-flowering treatment (N4), this trait was caused by a higher grain N concentration in the high-N pre-flowering treatment (N10).

The relation between GY and grain N concentration was also observed at two intermediate stages of grain development. At GS65+250 DD (Fig 1A) and at GS65+450 DD (Fig 1B), there was no significant genotype effect on GY, whereas grain N concentration was already significantly higher in Renan across all treatments (p<0.001). These results show that differences between the two genotypes in grain N concentration observed under N10 are likely to have been established early in grain development. Oppositely, the differences in grain yield observed under N4 occurred at a later stage. Pre-flowering N treatment effects on grain N concentration and GY were also already effective (p<0.001 and p = 0.003, respectively), revealing that for Récital, the N4 pre-flowering treatment led to higher grain N concentration than N10 early during grain filling.

### Post-flowering N uptake and N remobilisation

**Fig 2:**
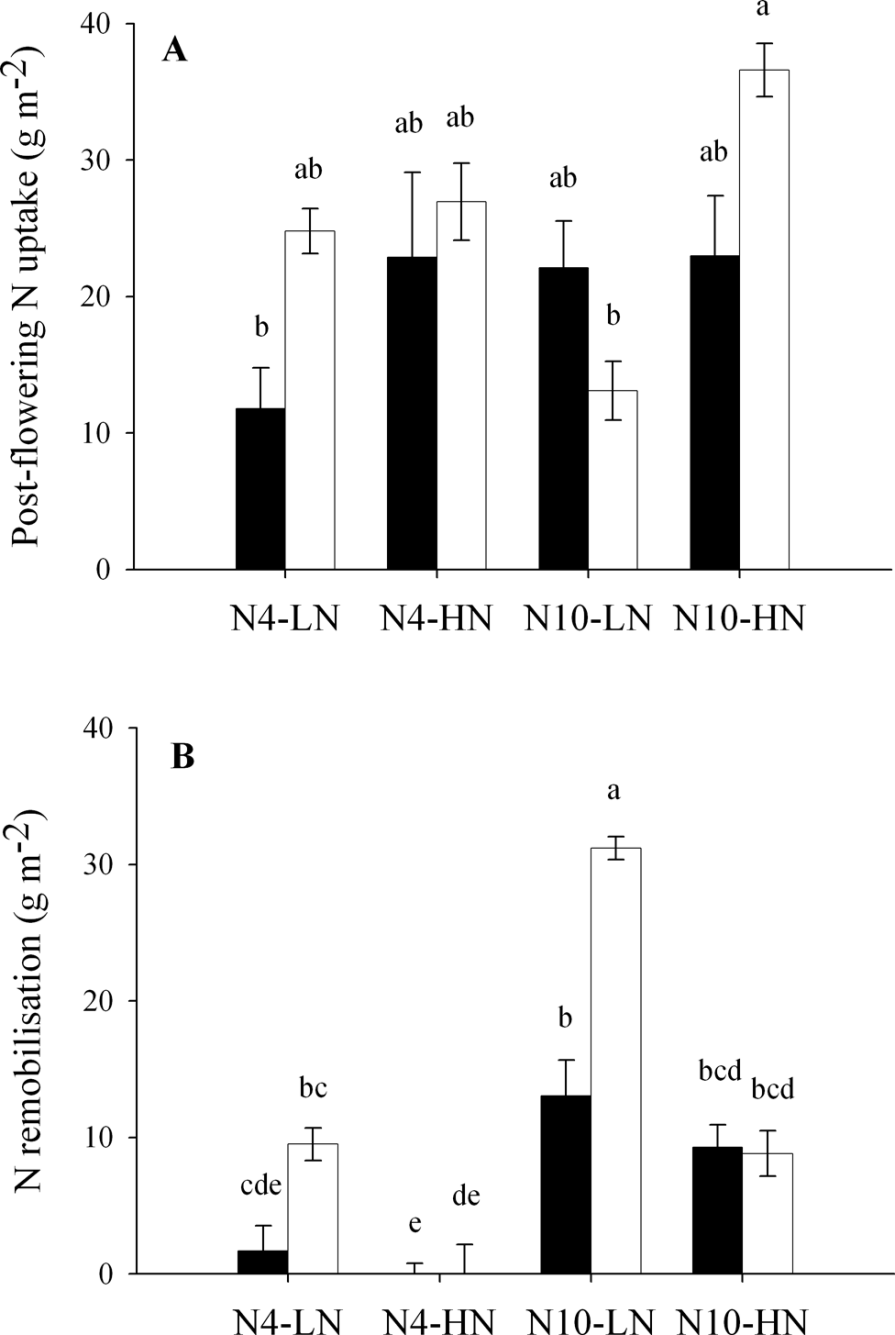
**Post-flowering N uptake (A) and N remobilisation (B) for Récital (black circles) and Renan (white circles) exposed to two contrasting pre-flowering nitrate treatments N4 and N10 combined with two contrasting post-flowering nitrate treatments LN and HN.** Values are the means of five biological replicates ± 1 standard error. Statistical groups are given by post-ANOVA Tukey HSD test for α = 0.05.

Post-flowering N uptake (PANU) ranged between 11 and 36 g m^-^² (Fig 2A), representing from 30 to 100% of total grain N at maturity. In the case of the HN post-flowering treatment, PANU represents the equivalent of 71 to 100% of total grain N. The Anova reveals a significant genotypic effect, with higher PANU levels in Renan (p = 0.04), and a strong post-flowering N treatment effect (p<0.001) showing that the HN treatment led to overall increases in PANU. However, a third-order interaction, genotype × post-flowering N treatment × pre-flowering N treatment (p = 0.003) emphasises that this trait is responsive to complex interaction effects, so the main factor effects should be interpreted cautiously. The main point to highlight for PANU is the marked difference observed between LN (12 g m^-^²) and HN (36 g m^-^²) post-flowering treatments in Renan plants subjected to the N10 pre-flowering treatment. This was clearly highly influenced by N availability during the post-flowering period.

In this study, it was found that PANU was not significantly correlated with N remobilisation (p = 0.458), which contrasts with significant and negative correlations reported from experiments carried out under field conditions that focused on genotypic effects [5,16,23]. Remobilisation of N from vegetative parts to the grain varied between 0 and 31 g m^-2^ (Fig 2B), representing from 0 to 70% of total grain N. In the case of HN post-flowering treatment, remobilisation always represented less than 29% of total grain N. Anova revealed that N remobilisation is a complex trait, impacted by all three main factors (genotype, p<0.001; pre-flowering N treatment, p<0.001; post-flowering N treatment, p<0.001) and interaction terms between genotype and post-flowering N treatment (p<0.001), together with a third-order interaction (p = 0.038). Overall, Renan remobilised more than Récital, which is mostly explained by the high values observed for Renan plants exposed to N10-LN and N4-LN treatments. Remobilisation was also generally favoured in the N10 pre-flowering treatment and LN post-flowering treatment, leading to high N stocks in vegetative parts at flowering and low N availability during the post-flowering period, respectively. Lastly, the interaction between genotype and post-flowering N treatment effects reflects the higher capacity of Renan to increase its remobilisation when exposed to the LN post-flowering N treatment.

### Relation between grain N concentration and early PANU

**Fig 3:**
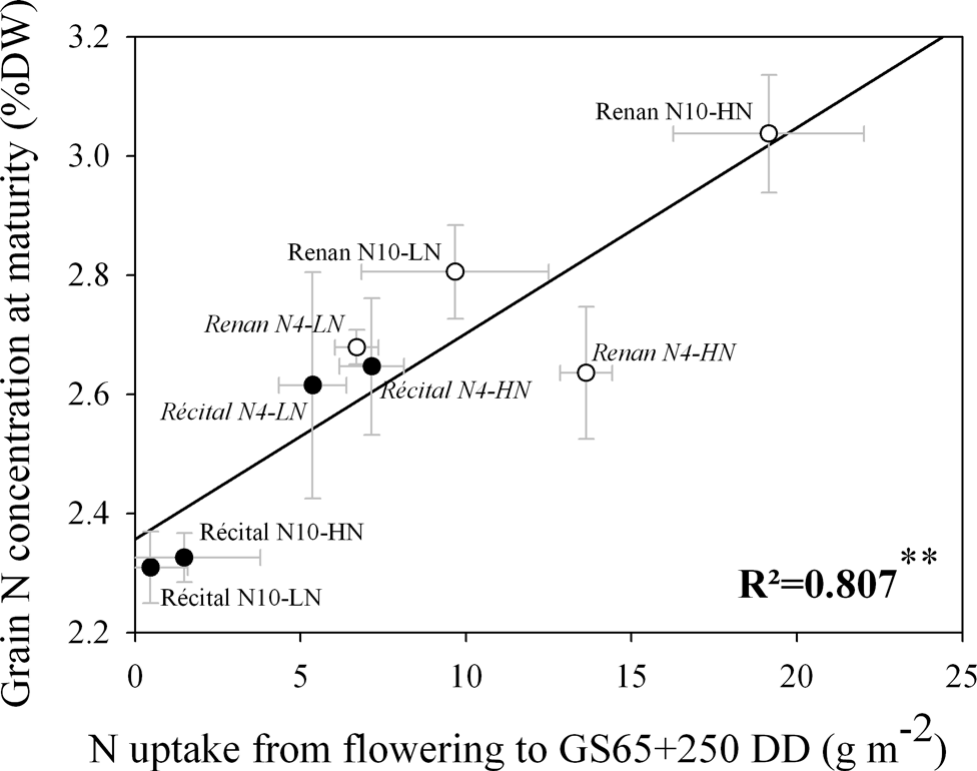
Relation between N uptake from flowering to GS65+250 DD and grain N concentration at maturity for Récital (black circles) and Renan (white circles) exposed to two contrasting pre-flowering nitrate treatments N4 (italic labels) and N10 (regular labels) combined with two contrasting post-flowering nitrate treatments LN and HN. Values are the means of five biological replicates ± 1 standard error.

In this study, early PANU occurring from GS65 to GS65+250 DD represents a highly variable part of total PANU, ranging between 2% and 74% depending on genotype and N treatment. This early PANU was positively correlated with grain N concentration at maturity (p = 0.002) (Fig 3), as well as with grain N concentration measured at GS65+250 DD (p = 0.040), and at GS65+450 DD (p = 0.002) (S1 Fig). Among all measured traits in this study, early PANU was the only one which displayed a significant correlation with grain N concentration at maturity. Oppositely, total PANU measured at maturity was not significantly correlated with grain N concentration (p = 0.414).

### Physiological and molecular determination of early PANU

**Fig 4:**
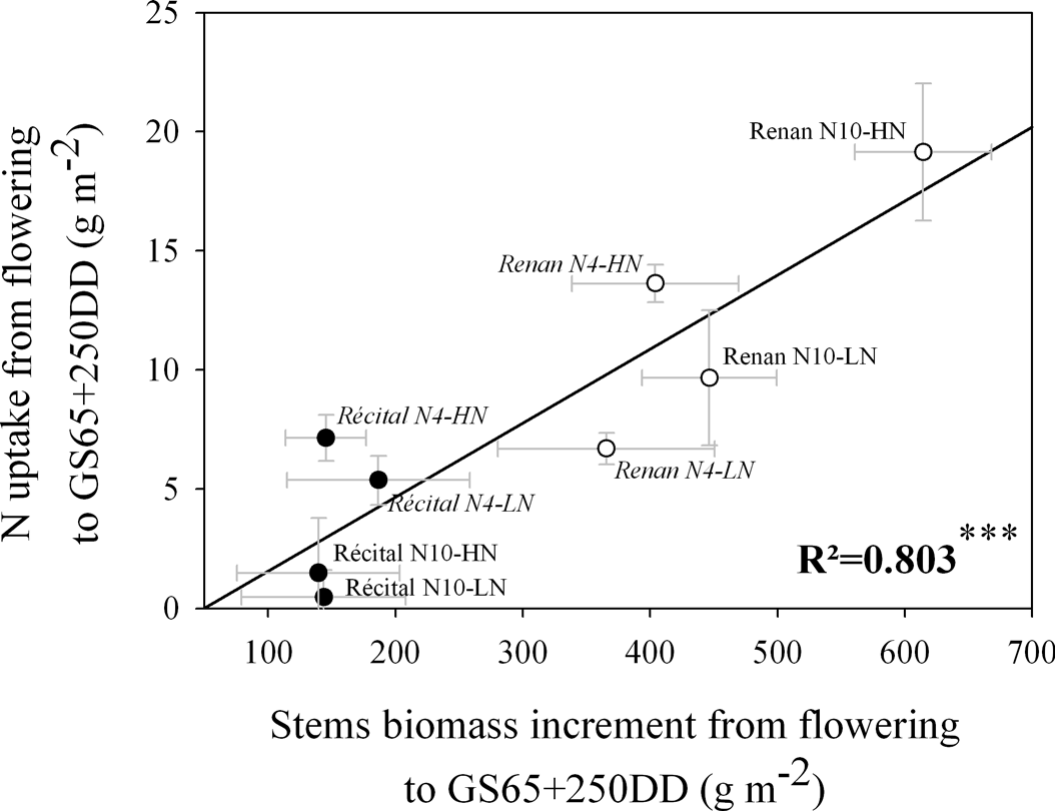
Relation between stem biomass increment and N uptake from flowering to GS65+250 DD for Récital (black circles) and Renan (white circles) exposed to two contrasting pre-flowering nitrate treatments N4 (italic labels) and N10 (regular labels) combined with two contrasting post-flowering nitrate treatments LN and HN. Values are the means of five biological replicates ± 1 standard error.

At a physiological level, early PANU at GS65+250 DD was positively correlated with the stem biomass increment between GS65 and GS65+250 DD (r² = 0.80; p = 0.003) (Fig 4); the stem biomass increment from GS65 to GS65+250 DD being itself under the influence of a genotypic effect (p<0.001) with higher levels observed for Renan.

**Fig 5:**
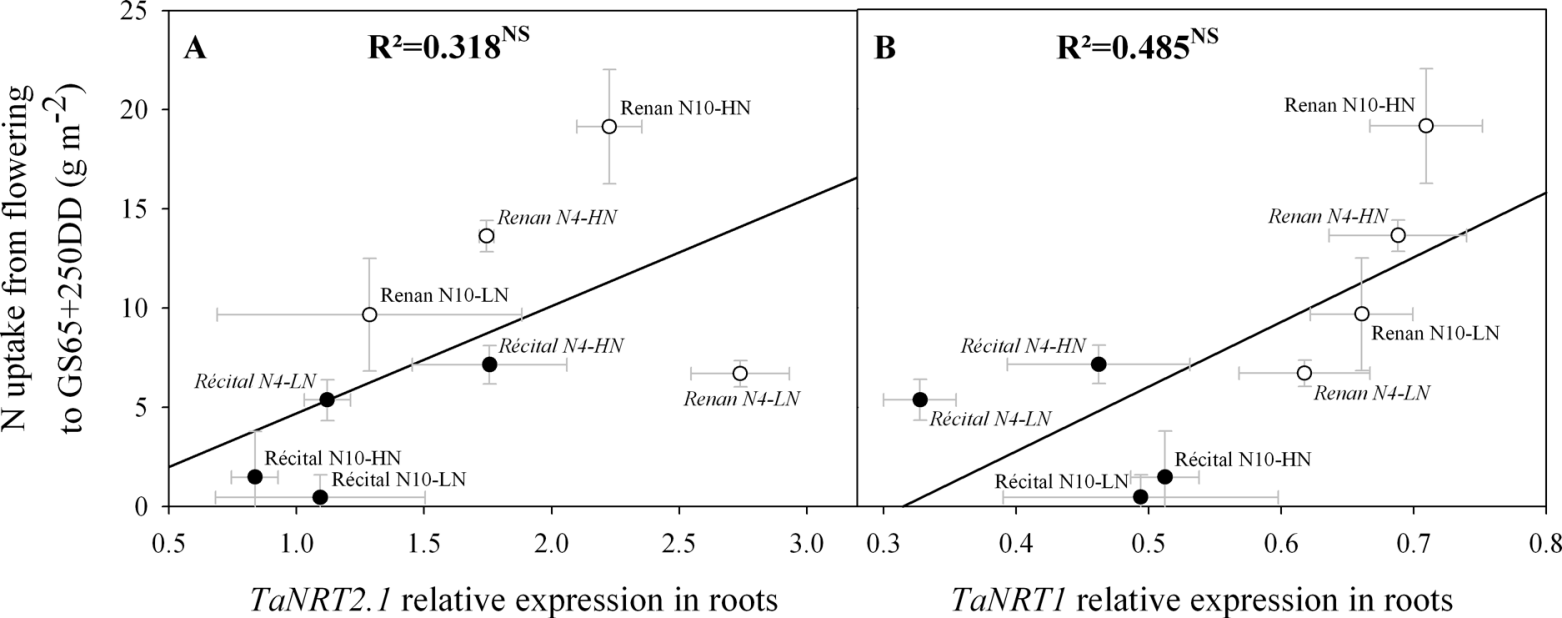
Relations between expression levels of genes coding for root nitrate transporters and N uptake from flowering to GS65+250 DD. Relations are for *TaNRT2*.*1* relative expression in roots at GS65+250 DD (A) and *TaNRT1* relative expression in roots at GS65+250 DD (B). Values are for Récital (black circles) and Renan (white circles) exposed to two contrasting pre-flowering nitrate treatments N4 (italic labels) and N10 (regular labels) combined with two contrasting post-flowering nitrate treatments LN and HN. Values are the means of five biological replicates ± 1 standard error.

**Fig 6:**
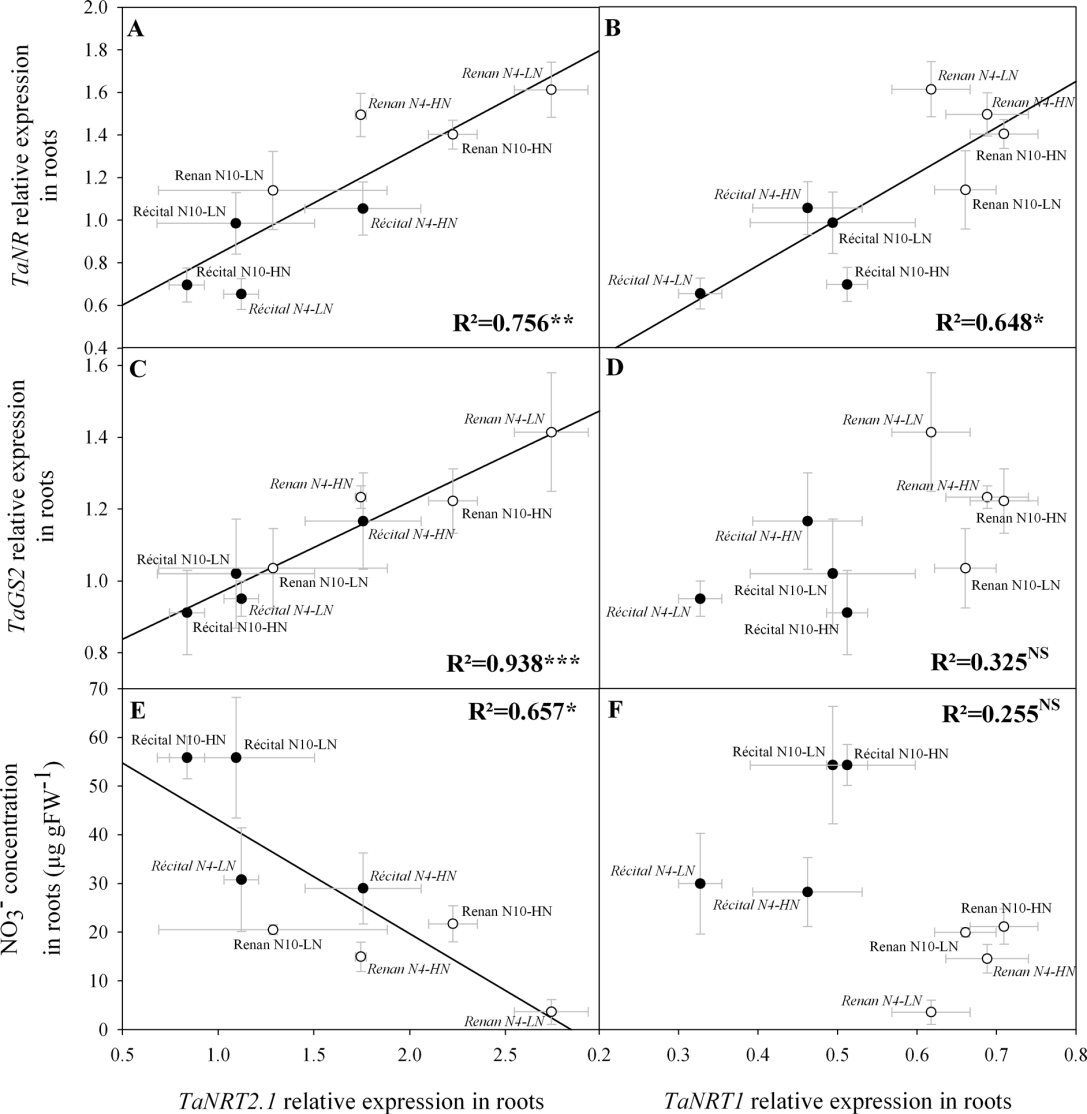
Relations between relative expression levels of genes coding for root nitrate transporters and relative expression levels of genes involved in nitrate assimilation, or nitrate concentration in roots at GS65+250 DD. Relations are for *TaNRT2*.*1* and *TaNR* (A), *TaNRT2*.*1* and *TaGS2* (C), *TaNRT2*.*1* and root NO_3_^-^ concentration (E), *TaNRT1* and *TaNR* (B), *TaNRT1* and *TaGS2* (D), *TaNRT1* and root NO_3_^-^ concentration (F). Values are for Récital (black circles) and Renan (white circles) exposed to two contrasting pre-flowering nitrate treatments N4 (italic labels) and N10 (regular labels) combined with two contrasting post-flowering nitrate treatments LN and HN. Values are the means of five biological replicates ± 1 standard error.

At a molecular scale, although not statistically significant, PANU at GS65+250 DD was positively correlated with both the expression of the NO_3_^-^ high-affinity transporter *TaNRT2*.*1* (p = 0.145) (Fig 5A) and that of the low-affinity transporter *TaNRT1* (p = 0.055) (Fig 5B). At GS65+250 DD, *TaNRT2*.*1* expression levels were significantly correlated with those of *TaNR* (Fig 6A) and *TaGS2* (Fig 6C). At the same developmental stage, *TaNRT1* expression levels correlated positively with those of *TaNR* (Fig 6B), but were not correlated with those of *TaGS2* (Fig 6D). Genes coding for root NO_3_^-^ transporters as well as genes coding for NR and GS2 exhibited higher expression levels in Renan than in Récital at GS65+250DD (p = 0.001, p<0.001, p<0.001, and p = 0.02, respectively for *TaNRT2*.*1*, *TaNRT1*, *TaNR*, and *TaGS2*).

Finally, *TaNRT2*.*1* expression and root NO_3_^-^ concentrations were negatively correlated at GS65+250 DD (p = 0.015) (Fig 6E), whereas no correlation was observed between *TaNRT1* expression and root NO_3_^-^ concentration (p = 0.202) (Fig 6F). Root NO_3_^-^ concentrations were significantly higher in Récital than in Renan (p<0.001). An effect of pre-flowering NO_3_^-^ treatment was also revealed by the Anova test, showing that the N10 treatment led to higher root NO_3_^-^ concentrations than the N4 treatment (p = 0.001).

## Discussion

### Growing conditions and choice of genotypes

The literature reveals that most studies of wheat root processes at a fine scale used plants grown under hydroponic conditions. The semi-hydroponic approach used here allows a fine control of N availability and it also allows to perform rapid changes to the effective N availability because of the low N-retention capacity of the substrate. Compared to field trials, these features are particularly useful in allowing independent control of the available N before and after flowering, without neglecting any genetic differences in terms of plant phenology.

In this study, we used an approach based on two contrasting NO_3_^-^ concentrations (4 and 10 mM) before flowering. The 10 mM NO_3_^-^ concentration is generally considered non-limiting for hydroponic wheat [27,34,52], while 4 mM NO_3_^-^ generates a significant N stress but without having drastic effects on canopy structure in terms of tiller density and dynamics [27]. Clearly, our objective of generating contrasting N statuses at flowering was met as shown by the significant differences between N4 and N10 for all the physiological traits measured at flowering (Table 1). After flowering, plants from each pre-flowering treatment were subjected to either a high-N (10 mM; HN) or a low-N (4 mM; LN) post-flowering N treatment in order to observe the independent effects of N availability before and after flowering. Although N status at flowering had large effects on GY, post-flowering N availability effects were lower than expected on GPC. This shows that even if the N4 treatment was clearly limiting for N during the pre-flowering period, the LN post-flowering treatment was not highly N deficient. However, these contrasting treatments allow significant differences in total grain N at maturity to be created, a circumstance particularly favourable for research into GPD determinism. To this aim, our strategy was to use two significantly contrasting genotypes for GPD based on an earlier robust characterisation of this trait in field studies [10,23]. The experimental design used in this study, comprising only two genotypes, did not allow calculating GPD values for Récital and Renan. However, genotypic ranking for GPD can be reasonably estimated by a ranking for protein yield [7,53]. Under our conditions, Renan clearly exhibited larger grain N values than Récital, showing that the genetic component implied in protein yield was expressed under our controlled conditions in a way similar to in the field [23,42,54]. These results suggest that GPD may be a sufficiently robust genetic character to be expressed even under such different growing conditions.

### GPC at maturity is correlated with early PANU at GS65+250 DD

Under field conditions, numerous studies have highlighted that PANU has a larger effect on grain N than on yield [5,14,24–26]. In the present study, although no correlation was observed between total PANU and GPC (r² = 0.11; p = 0.414), a strong correlation was found between GPC and early PANU (e.g. PANU calculated only between flowering and GS65 + 250 DD). This result, although only correlative, suggests that this trait could be a strong driver of final GPC. Quantitatively, early PANU represented on average 36% of total PANU. In a study based on four spring wheat genotypes grown hydroponically under condition of gradual decrease of N availability during post-flowering period mimicking field conditions, Oscarson *et al*. [19] observed an even higher value, with about 50% of total PANU occurring before the end of grain cell division. Hence, early PANU may represent a large part of total PANU under field conditions because of the various environmental limits to PANU occurring later during grain filling, water and N stresses in particular. This could be the reason for the stronger effect of total PANU on GPC observed under field conditions than in the present study. Taken together, these results plead for strong effect of early PANU on GPC, accounting both for existing genetic and environmental variabilities.

Datasets that allow us to temporally dissect the relationship established over the entire post-flowering period are scarce. Under non-limiting conditions for N, in hydroponic culture, Taulemesse *et al*. [27] highlighted that PANU exhibits a marked dynamic during the post-flowering period. Further, these authors showed that PANU occurring during the early post-flowering phase (grain cell division) was strongly impacted by plant N status at flowering, whereas that occurring during the later post-flowering phase (active phase of grain N filling) was hypothesised to be mainly controlled by N demand exerted by grain growth. Clearly, our results suggest that the N taken up after flowering has a differential effect on GPC depending of the timing at which it occurs during the post-flowering period. Supporting this idea, in a study based on a high GPC genotype grown hydroponically, Oscarson *et al*. [20] have shown that the timing of short extra applications of N during the post-flowering period impacts GPC for N-limited plants. However, it is difficult to know whether the results of Oscarson *et al*. can be transposed to non-deficient plants where GY is not impacted by N starvation during the post-flowering period as in our study. With a different approach based on a multi-local study under field conditions on mapping populations, Bogard *et al*. [23] reported that GPD measured at GS65+250 DD is well correlated with GPD measured at maturity (r² = 0.50), possibly indicating an early determinism of this trait. As GPD was strongly correlated with genotype capacity to take up N after flowering independently of the level of N uptake before flowering, these authors hypothesised that early PANU could play a strong role in GPD. This hypothesis is in accordance with our results which show that a high early PANU is associated with a high GPC under our controlled conditions, independently of GY level.

### Genetic variability for early PANU regulation

Quantitatively, early PANU represents a highly variable component of total grain N at maturity, ranging from 1% to 51% depending on genotype and N treatment. On average, early PANU was higher for Renan than for Récital (12.28 g m^-2^ and 3.6 g m^-2^, respectively). In addition to these quantitative variations, the two genotypes displayed contrasting controls of early PANU (S2 Fig). On the one hand, in Récital, the ratio of early PANU over total grain N at maturity was influenced mainly by plant N status at flowering (*i*.*e*. showing a strong effect of pre-flowering N treatment) with values of about 41% for plants exposed to the N4 pre-flowering treatment, and less than 7% for plants exposed to the N10 one, with no effect of the post-flowering N treatment. Comparable results have already been described for Récital, with higher early PANU observed for plants having low N status at flowering [27]. On the other hand, in Renan, this ratio was impacted mainly by post-flowering N availability, with values around 20% for plants exposed to the LN post-flowering treatment, compared with about 45% for plants exposed to the HN one, while the effect of pre-flowering N treatment was negligible. This argues strongly for genotypic differences in the control of early PANU.

The regulation of N uptake is under the control of complex mechanisms. Earlier studies have shown that N uptake is driven not only by soil N availability but also by plant N demand [4,55–57]. Under hydroponic conditions, plant N uptake occurs until late in plant development [19–22,27,58], and roots have the ability to take up N even when the concentration in the medium is extremely low [19]. Under such conditions, plant N demand could play a central role in N uptake variability. Accordingly, the early PANU observed in Récital was not significantly affected by post-flowering N availability, while the effect of plant N status at flowering was dominant. However, the observations in Renan suggest plant N status at flowering have less impact on early PANU for this genotype because early PANU was clearly limited by N availability for both N statuses at flowering under LN post-flowering condition. Despite the fact that Renan and Récital had roughly comparable NNI at flowering, their N uptakes differed during the days following flowering, particularly for plants exposed to the N10 pre-flowering treatment. This suggests that NNI, which is a valuable integrative indicator of limitation of carbon acquisition by plant N concentration during vegetative growth [46], does not allow the assessment of post-flowering N uptake as genetic variability exists at particular NNI values. These findings argue for the existence of a genetic difference in N satiety at similar NNI, which could be defined as the maximum level of plant N accumulation corresponding to a state of plant N saturation. Genotypic variability for N satiety may thus be related to genotypic deviations from the “maximum N dilution curve” described by Justes *et al*. [46]. Hence, N satiety may influence the genotypic capacity to take up luxury levels of N, *i*.*e*. amounts not strictly necessary for growth but possibly beneficial to GPC. Genetic differences for N satiety have previously been hypothesised to be involved in the determination of GPD [23], and could also explain the reasons for the higher sensibility to N starvation of Récital observed previously [59].

### Genetic variability for early PANU is linked to vegetative growth after flowering

As noted above, internal regulatory mechanisms of N uptake are driven mainly by physiological parameters such as organ growth and the N concentration of already-formed organs. Early PANU occurs during a period when growing organs represent only a moderate biomass accumulation, because grains have not yet reached a rapid growth phase and the photosynthetic apparatus is already fully developed. The grain biomass increment from GS65 to GS65+250 DD does not explain early PANU, as these two traits were not significantly correlated in the present study (p = 0.261). This finding is in accordance with previous results obtained by Taulemesse *et al*. [27] in Récital which rather suggest a major role for plant N status in N uptake at this developmental stage. In the framework of model development for grain protein concentration in wheat, Martre *et al*. [60] suggested that the rate of N uptake after flowering could be limited by the N storage capacity of the stem. In this sense, some studies have shown that stem biomass may continue to increase during about one week after flowering, especially through peduncle elongation [61,62]. Potentially, the growth of this structural organ could create a sink demand for N, thus influencing N uptake, especially as stem biomass increments from GS65 to GS65+250 DD exceeded grain biomass accumulation for some genotype x N treatment combinations in the present study. As a result, early PANU displayed a positive correlation with the stem biomass increment from GS65 to GS65+250DD (Fig 4) but not with stem N concentration variation during the same phase (p = 0.946). Clearly, the correlative approach used in the present study does not provide any causal demonstration of the link between the two traits. However, the high levels of NNI measured at flowering do seem to indicate that plant growth during the early post-flowering phase was not limited by plant N under our conditions, with values always higher than 1. This suggests that observed genetic differences for stem growth after flowering were probably not a consequence of N uptake, but were more likely partly causal. Regulation of stem growth after flowering could thus be involved in grain N concentration determination.

### Molecular determination of early PANU

Genes implicated in plant N uptake and N assimilation network have been described largely in model species such as *A*. *thaliana* [30–33]. Not all homologs of these genes have yet however been characterised in wheat, leading to an incomplete characterisation of N uptake through gene expression studies. Nevertheless, expression quantification of some key genes involved at different levels of N metabolism allows the establishment of a good overview of the N assimilation process. In this study, we observed no significant correlation between early PANU and root N transporter expression, either for *TaNRT2*.*1* or *TaNRT1*. This result differs from that of a previous study [27] showing a significant correlation between PANU and *TaNRT2*.*1* expression level in wheat. Similarly, in maize, Garnett *et al*. [63] showed that *ZmNRT2*.*1* and *ZmNRT2*.*2* expression patterns correlated well with the N uptake pattern. The methodology used in the present study to calculate early PANU may partly explain this lack of significant correlation, as PANU was calculated based on total plant N differences between two sampling dates (and therefore integrating plant functioning over the whole period) while expression results were based on instantaneous measurements. We believe this may have reduced the accuracy of the relationship which nevertheless shows a positive trend (Fig 5A).

In addition, the relation between *TaNRT2*.*1* expression at GS65+250 DD and early PANU was significantly positive (p = 0.007) after exclusion of Renan N4-LN. This treatment x Genotype combination exhibited the highest *TaNRT2*.*1* expression level but a very low N uptake. In this case, a consequent N demand that cannot be fulfilled under LN conditions can be strongly hypothesised. The positive correlation between early PANU and *TaNRT2*.*1* expression levels at GS65+250 DD without Renan N4-LN suggests that *TaNRT2*.*1* could have played an important role in early PANU, although its expression levels did not explain N uptake when N demand was not satisfied. This assumption is in accordance with recent studies suggesting a preponderant involvement of HATS on wheat nitrogen uptake independently of NO_3_^-^ concentration in the external medium [38,39].

This assumption is also strengthened by the observation that *TaNRT2*.*1* expression was in turn significantly correlated with *TaNR* and *TaGS2* expression levels at GS65+250 DD, suggesting a consistency between N uptake, N reduction and N assimilation genes (Fig 6). A synchronous regulation of genes coding for N transport, reduction and assimilation against NO_3_^-^ environment has been substantially described in a range of species such as *A*. *thaliana* [64], *Oriza sativa* [65], and *Zea mays* [66–69]. However, the regulation of N uptake and N assimilation systems at the molecular level has not clearly been established, as at least two putative regulatory signals have been proposed for N uptake regulation. The first is circulating amino-acids such as glutamine [70–72] and the second is NO_3_^-^ itself [73–75]. In Taulemesse *et al*. [27] and in the present study, *TaNRT2*.*1* expression levels at GS65+250 DD were negatively correlated with NO_3_^-^ concentration in roots, suggesting that NO_3_^-^ concentration could be an internal marker of plant N demand with a putative direct or indirect regulatory role on genes coding for N metabolism. Similarly, *TaNR* and *TaGS2* expression levels were negatively correlated with root NO_3_^-^ concentration (S3 Fig). The hypothesis identifying NO_3_^-^ concentration in roots as a marker of plant N demand is also supported by physiological measurements, as NO_3_^-^ levels in roots were significantly lower in N4 than in N10 at flowering independently of genotype. The rapid increase in NO_3_^-^ level in roots after the switch from N4 to HN tends to corroborate this assumption. The putative role of NO_3_^-^ concentration in roots as a marker of plant N demand is also implied by genotypic differences. Indeed, the higher levels of early PANU observed in Renan were accompanied by lower root NO_3_^-^ concentrations in this genotype than in Récital, regardless of the pre-flowering N treatment (S4 Fig).

Excluding Renan N4-LN, the relation between NO_3_^-^ concentration and early PANU became significant (p = 0.018; S5 Fig). The outlier nature of Renan N4-LN is probably a result of the same factors previously suggested for the relation between *TaNRT2*.*1* expression level and early PANU. Genetic effects on early PANU could thus be based on differences in NO_3_^-^ concentration in roots, despite comparable NNI, revealing different N satiety levels.

### Putative influence of satiety level on the balance between PANU and N remobilisation

In the present study, PANU and N remobilisation were not significantly correlated (p = 0.458) when considering the two genotypes and the four N conditions. The absence of correlation was probably influenced by the contrasting N environments used in both the pre-flowering and the post-flowering periods, but was also a result of a genotypic effect. Renan showed both higher PANU and N remobilisation than Récital with all treatments combined (Fig 2), showing that Récital did not compensate for its lower N uptake by N remobilisation. The N remobilisation under LN treatment in Renan was generally higher, leading to a higher NHI of this genotype than Récital when exposed to low N availability during the post-flowering period (Table 2). Under field conditions, the low capacity of Récital to achieve high remobilisation levels has already been observed [14]. These results seem to indicate that Renan has both a higher capacity to take up N when N is available and a lower threshold for N remobilisation than Récital. Based on the fact that remobilisation is involved when N uptake cannot meet N demand, it is plausible that the specific satiety level of each genotype influences the triggering of this phenomenon. According to this, despite comparable NNI levels, a variety having a high luxury N demand must have more difficulty taking up enough N to be satiated than a variety having a low luxury N demand, which may precipitate the onset of senescence. Thus, as root NO_3_^-^ concentration seems to be a reliable indicator of plant N demand, cultivars presenting low NO_3_^-^ concentrations in their roots even at high NNI, could be both more effective in N uptake when N is available, and better able to use N stored in vegetative parts when the availability of N is insufficient during grain filling.

## Conclusions

This study aimed to identify genetic differences in the establishment of GPC in order to better understand the genetic bases of GPD. Based on the behavior of two genotypes having strongly contrasting GPC grown under controlled conditions with varying NO_3_^-^ availabilities, we show that GPC is positively correlated with early PANU from GS65 to GS65+250 DD independently of GY level. At a physiological level, the study suggests that early PANU could be impacted by stem biomass increment during early growth stages following flowering, although the regulatory mechanisms of this are unknown. At a molecular scale, the negative correlation between root NO_3_^-^ concentration and N network genes expression levels suggests that root NO_3_^-^ concentration is a good candidate for evaluating instantaneous plant N demand, and may provide valuable information on genotype satiety level when measured at high NNI. Although these findings have still to be validated with a larger number of genotypes, this study attempts to open new research routes to develop a better understanding of the physiological and molecular bases of genetic determination of grain N concentration involved in GPD. On the basis of our results, genotypes having a low NO_3_^-^ concentration in the roots, even at high NNI levels, may be interesting subjects for breeding as they have a tendency to accumulate more N both at flowering and during the early post-flowering growth stages, but they also seem more able to remobilise N, when its availability is reduced during grain filling.

## Supporting Information

S1 Dataset Raw data used in the study. (XLSX)

S1 Fig Relation between N uptake from flowering to GS65+250 DD and grain N concentration at GS65+250 DD (A) or at GS65+450 DD (B).
(TIF)

S2 Fig Proportion of total grain N at maturity represented by N uptake from flowering to GS65+250 DD. (TIF)

S3 Fig Relations between nitrate concentration and *TaNR* relative expression (A) or between nitrate concentration and *TaGS2* relative expression (B) in roots at GS65+250 DD.
(TIF)

S4 Fig Nitrate concentration in roots at flowering and at GS65+250 DD. (TIF)

S5 Fig Relations between nitrate concentration in roots at GS65+250 DD and N uptake from flowering to GS65+250 DD. (TIF)

S1 Table Elemental composition of nutrient solutions adapted from Castle and Randall (1987). (PDF)
