# Supplementary figures and images for: Bread Wheat (Triticum aestivum L.) Grain Protein Concentration Is Related to Early Post-Flowering Nitrate Uptake under Putative Control of Plant Satiety Level
Source: PLoS One. 2016 Feb 17;11(2):e0149668. doi: 10.1371/journal.pone.0149668 (PMC4757577; doi:10.1371/journal.pone.0149668)

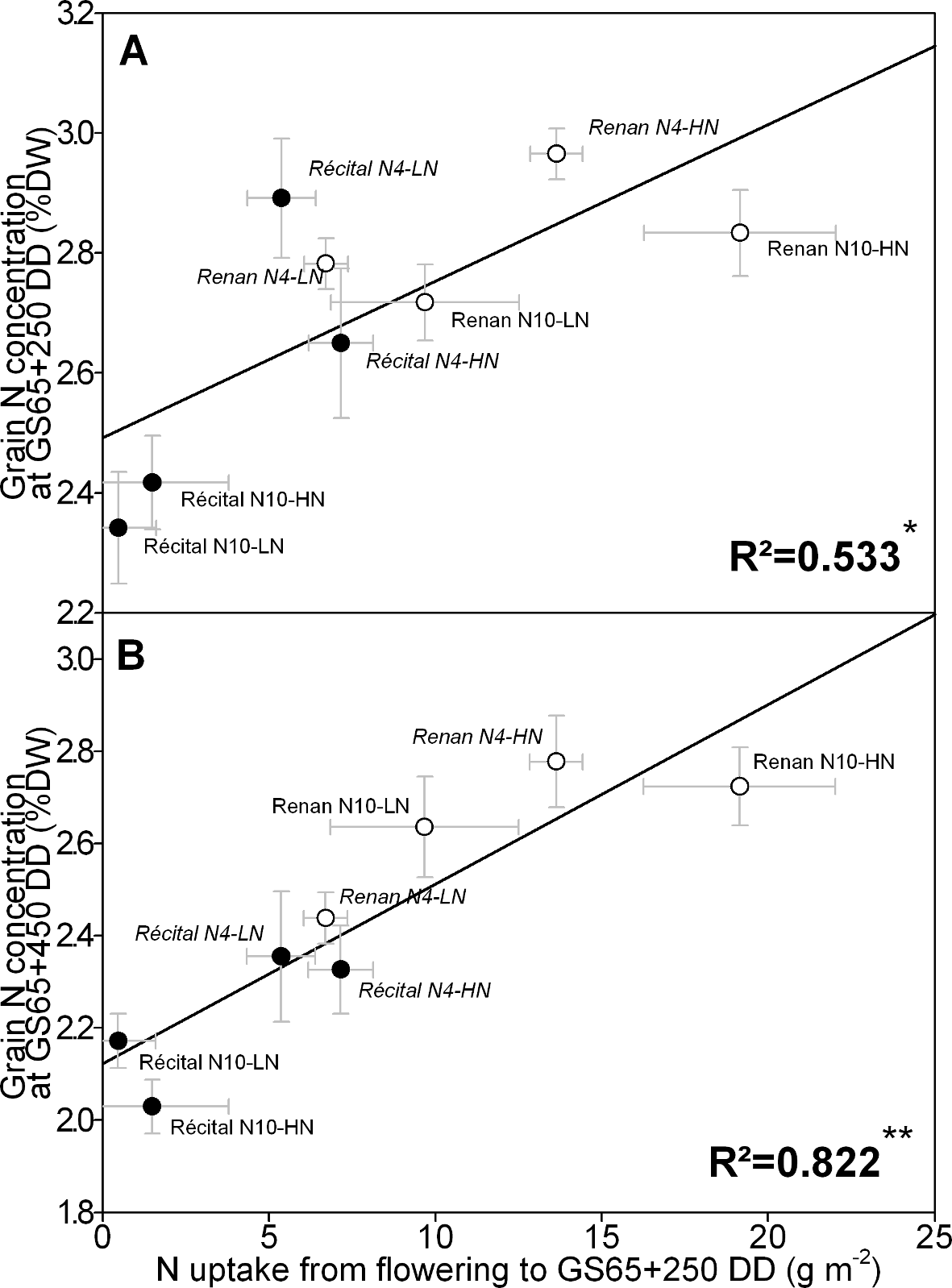

Supplement: S1 Fig — Relation between N uptake from flowering to GS65+250 DD and grain N concentration at GS65+250 DD (A) or at GS65+450 DD (B). (TIF) [file pone.0149668.s002.TIF]

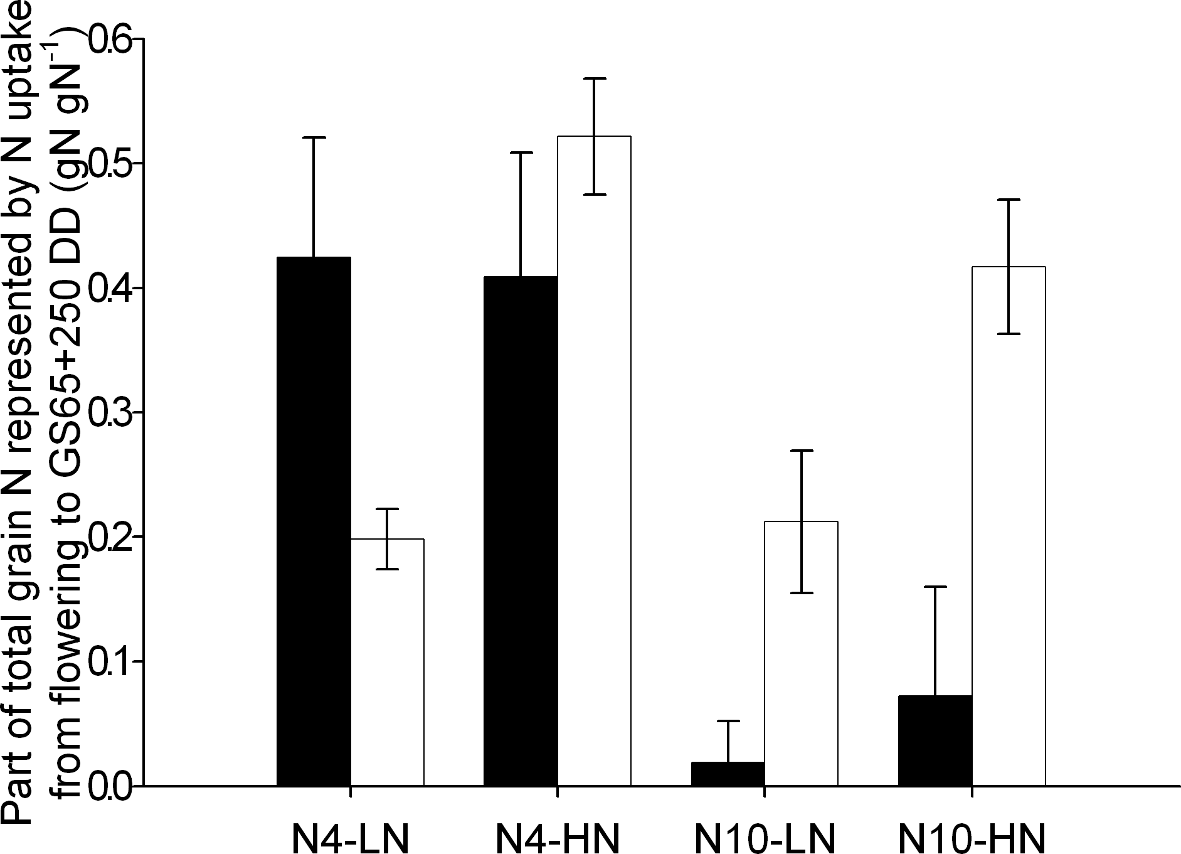

Supplement: S2 Fig — (TIF) [file pone.0149668.s003.TIF]

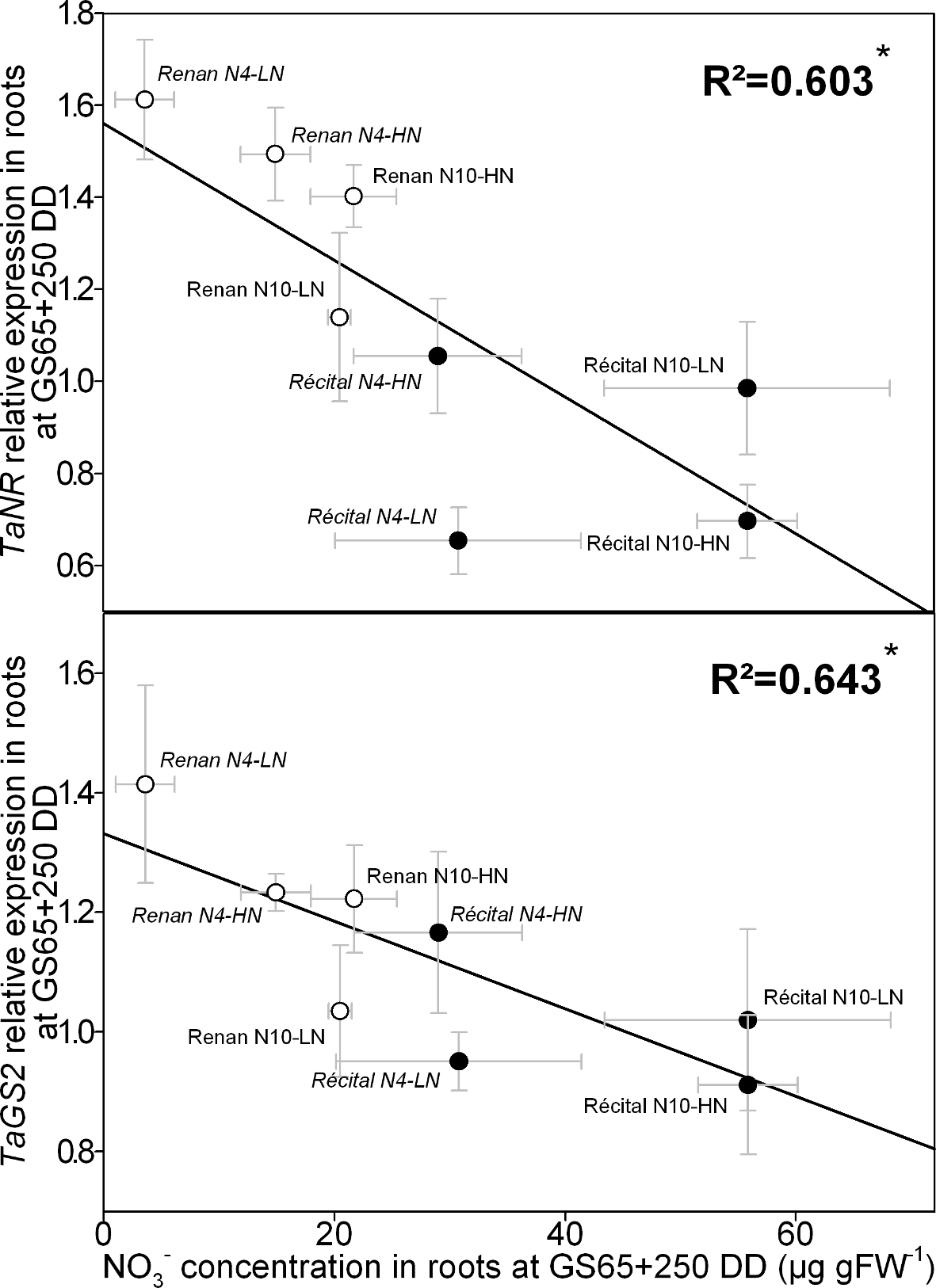

Supplement: S3 Fig — Relations between nitrate concentration and TaNR relative expression (A) or between nitrate concentration and TaGS2 relative expression (B) in roots at GS65+250 DD. (TIF) [file pone.0149668.s004.TIF]

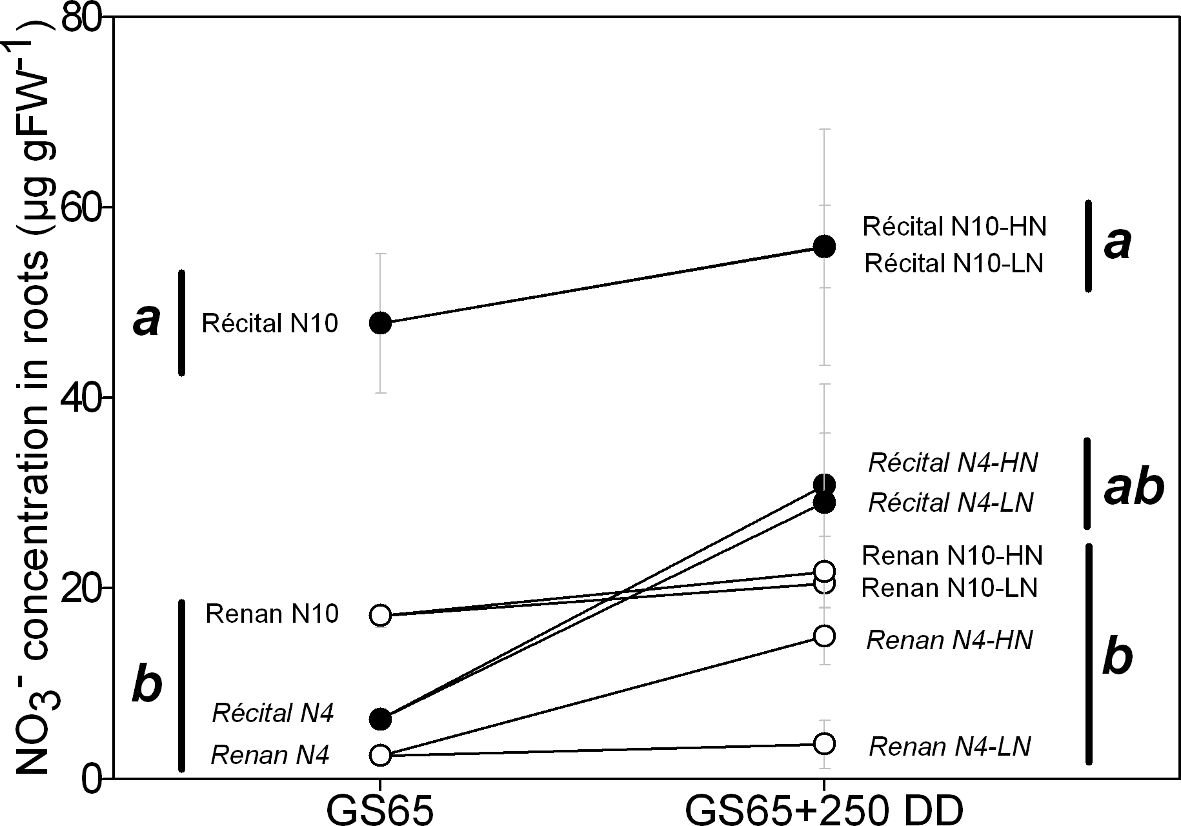

Supplement: S4 Fig — (TIF) [file pone.0149668.s005.TIF]

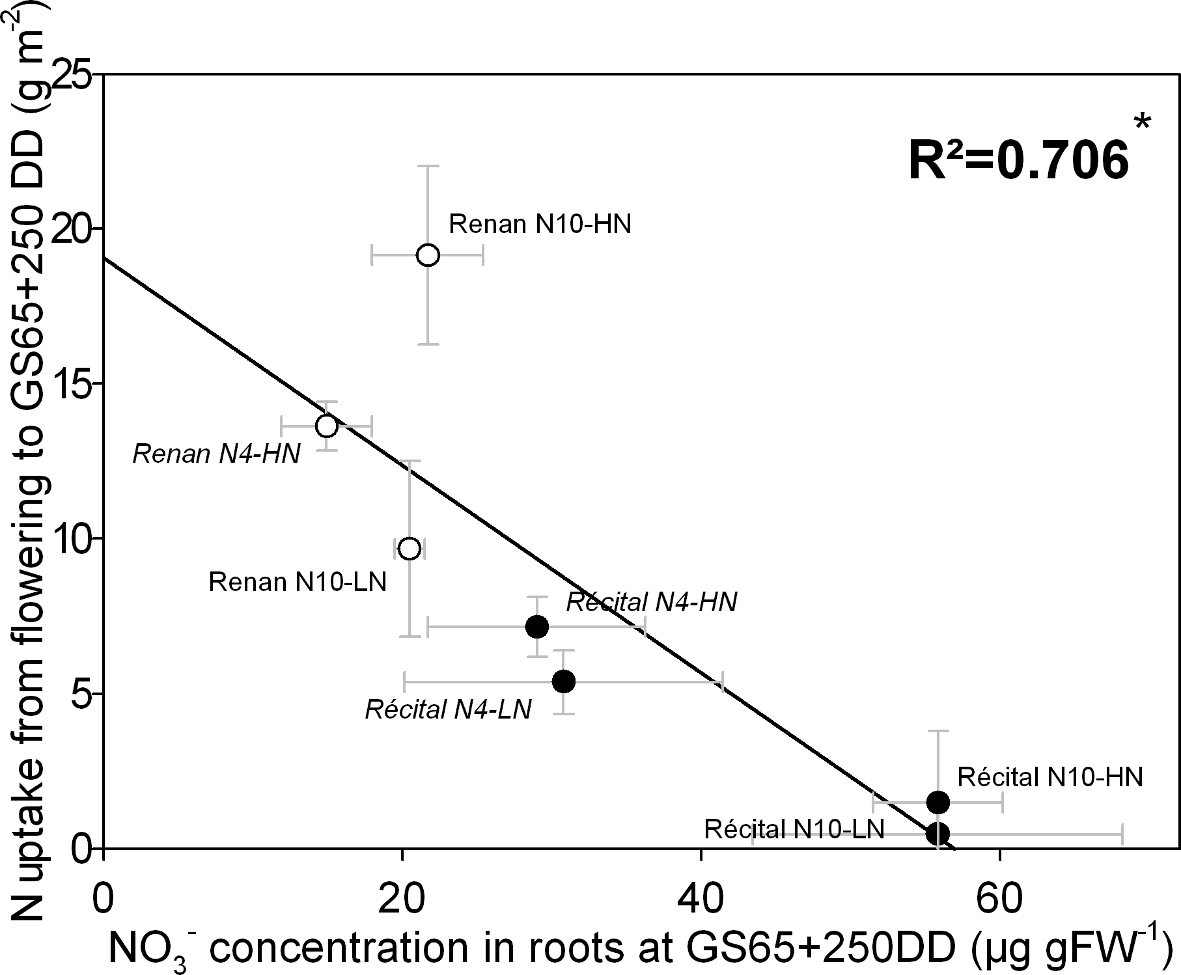

Supplement: S5 Fig — (TIF) [file pone.0149668.s006.TIF]
